# Evaluation of prediction errors in nine intraocular lens calculation formulas using an explainable machine learning model

**DOI:** 10.1186/s12886-024-03801-2

**Published:** 2024-12-19

**Authors:** Richul Oh, Joo Youn Oh, Hyuk Jin Choi, Mee Kum Kim, Chang Ho Yoon

**Affiliations:** 1 Department of Ophthalmology, Seoul National University Hospital, 101 Daehak-ro, Jongno-gu, Seoul, 03080 Korea; 2 Department of Ophthalmology, Seoul National University College of Medicine, Seoul, Korea; 3 Laboratory of Ocular Regenerative Medicine and Immunology (LORMI), Artificial Eye Center, Seoul National University Hospital Biomedical Research Institute, Seoul, Korea; 4 Department of Ophthalmology, Seoul National University Hospital Healthcare System Gangnam Center, Seoul, Korea

**Keywords:** LightGBM, Explainable artificial intelligence, Intraocular lens, Prediction error

## Abstract

**Background:**

The purpose of the study was to evaluate the relationship between prediction errors (PEs) and ocular biometric variables in cataract surgery using nine intraocular lens (IOL) formulas with an explainable machine learning model.

**Methods:**

We retrospectively analyzed the medical records of consecutive patients who underwent standard cataract surgery with a Tecnis 1-piece IOL (ZCB00) at a single center. We calculated predicted refraction using the following IOL formulas: Barrett Universal II (BUII), Cooke K6, EVO V2.0, Haigis, Hoffer QST, Holladay 1, Kane, SRK/T, and PEARL-DGS. We used a LightGBM-based machine learning model to evaluate the explanatory power of ocular biometric variables for PEs and assessed the relationship between PEs and ocular biometric variables using Shapley additive explanation (SHAP) values.

**Results:**

We included 1,430 eyes of 1,430 patients in the analysis. The SRK/T formula exhibited the highest R^2^ value (0.231) in the test set among the machine-learning models. In contrast, the Kane formula exhibited the lowest R^2^ value (0.021) in the test set, indicating that the model could explain only 2.1% of the PEs using ocular biometric variables. BUII, Cooke K6, EVO V2.0, Haigis, Hoffer QST, Holladay 1, PEARL-DGS formulas exhibited R^2^ values of 0.046, 0.025, 0.037, 0.194, 0.106, 0.191, and 0.058, respectively. Lower R^2^ values for the IOL formulas corresponded to smaller SHAP values.

**Conclusion:**

The explanatory power of currently used ocular biometric variables for PEs in new-generation formulas such as BUII, Cooke K6, EVO V2.0 and Kane is low, implying that these formulas are already optimized. Therefore, the introduction of new ocular biometric variables into IOL calculation formulas could potentially reduce PEs, enhancing the accuracy of surgical outcomes.

**Supplementary Information:**

The online version contains supplementary material available at 10.1186/s12886-024-03801-2.

## Significance

What was known before

What this study adds

Previous studies have evaluated the accuracy of intraocular lens power formulas for specific subgroups of patients according to the ocular biometric variables and provided valuable insights into the strengths and weaknesses of various formulas. However, the ocular biometric parameters showed significant associations with each other.

Compared with those of formally generated formulas, recent intraocular lens formulas yielded relatively stable outcomes across a wide range of ocular biometric variables. Ocular biometric variables have no significant effect on postoperative prediction errors.

**Supplementary Information:**

The online version contains supplementary material available at 10.1186/s12886-024-03801-2.

## Background

The primary goal of modern cataract surgery is to achieve optimal vision and predicted refraction. The prediction accuracy of postoperative refraction has increased significantly in recent years. The development of advanced optical technologies and intraocular lens (IOL) power calculation formulas have improved refractive outcomes. Conventional IOL formulas, such as the SRK/T [1], Haigis [2], and Barrett Universal II (BUII) [3] formulas, are based on theoretical lens calculations. More complex optics and effective lens position calculation for IOL power calculation have been incorporated in recently developed formulas, such as EVO 2.0 and Kane formulas [4]. 

Several studies have attempted to develop optimal IOL formulas for specific eye groups by classifying the eyes according to the axial length (AL) and comparing the refractive outcomes among the subgroups [5–9]. Other studies have revealed remarkable variations among the refractive outcomes of different subgroups according to the anterior chamber depth (ACD, measured from corneal epithelium to lens) [10, 11]. New-generation formulas demonstrate higher overall accuracy by incorporating more ocular biometric variables [12, 13]. Despite this advancement, there remains a need to further enhance IOL formulas for improved precision.

Ocular biometric variables show significant correlations with each other [14]. Thus, multiple ocular biometric factors affect the refractive outcomes of cataract surgery. Multivariable analysis must be conducted considering these parameters simultaneously to calculate the prediction errors (PEs), rather than attributing the PEs to individual biometric parameters.

In this study, we used a machine learning model to predict the refractive outcomes of nine IOL power formulas using ocular biometric variables to investigate the overall influence of ocular biometric variables on PE and compare the prediction outcomes of the formulas.

## Methods

This study was approved by the Institutional Review Board of the Seoul National University Hospital (SNUH; IRB No. 2112-132-1284) and adhered to the principles of the Declaration of Helsinki. The Institutional Review Board waived the requirement for obtaining written informed consent owing to the retrospective study design and anonymization of patient information.

### Study population

We retrospectively reviewed the medical records of patients who had undergone standard cataract surgery between August 1, 2018, and December 31, 2021. The inclusion criteria were as follows: (1) operation using Tecnis ZCB00 (Johnson & Johnson Vision Care, Inc., Santa Ana, CA, USA) IOL insertion in the bag, and (2) age of at least 19 years old. The exclusion criteria were as follows: (1) history of previous vitrectomy, corneal refractive surgery, or other corneal operation; (2) incidence of severe intraoperative or postoperative complication, such as zonular dialysis, posterior capsular rupture, or the use of capsular tension ring or iris retractor; (3) combined operation for the correction of glaucoma, pterygium, or vitrectomy; (4) cataract operation with large corneal or limbal incision or limbal relaxing incision; (5) absence of postoperative manifest refraction data; (6) postoperative best-corrected visual acuity (BCVA) of worse than < 20/40; (7) failure of ocular biometric examination; (8) inability to calculate IOL power owing to extreme refractive target. The first eye that underwent surgery was included if both eyes were eligible for inclusion.

### Preoperative ophthalmic evaluation

In accordance with the SNUH preoperative cataract examination protocol, all patients underwent a comprehensive ophthalmologic examination, which included BCVA assessment, slit-lamp biomicroscopy, dilated funduscopic examination, ocular biometric measurement (IOLMaster 700; Carl Zeiss, Germany), autokeratometry (KR-7100; Topcon, Japan), anterior segment topographic measurements (Orbscan II; Bausch and Lomb, USA), specular microscopy (NSP-9900; Konan Medical, Japan), optical coherence tomography (Heidelberg Spectralis; Heidelberg Engineering, Germany), and ultra-widefield fundus photography (Optos California; Optos, USA), preoperatively.

### Surgical procedures

All cataract surgeries were performed by the experienced surgeons at SNUH, as described below. The procedures were as follows: 2.2-mm or 2.75-mm small, clear corneal incision, continuous curvilinear capsulorrhexis, phacoemulsification of the crystalline lens, and implantation of the IOL in the capsular bag. The corneal incision was made at a temporal or superior location at the discretion of the surgeon.

### Prediction of postoperative refractive errors

We predicted the postoperative refractive errors using nine IOL power calculation formulas: Barrett Universal II (BUII), Cooke K6, EVO V2.0, Haigis, Hoffer QST, Holladay 1, Kane, SRK/T, and PEARL-DGS. We used the manufacturers’ and IOL calculators’ recommended constants available from online (https://www.iolcon.org/lensesTable.php; accessed April 5, 2024) for each IOL formula. The A constant of the IOL used was 119.3. The Haigis constants for a0, a1, and a2 were − 1.302, 0.210, and 0.251, respectively. The SRK/T formula requires the A constant for the IOL, corneal power, and AL. An investigator (RO) manually entered the data into online calculators for the BUII, Cooke K6, EVO V2.0, Hoffer-QST, Kane, and PEARL-DGS formulas (https://calc.apacrs.org/barrett_universal2105/, https://cookeformula.com/, https://www.evoiolcalculator.com/calculator.aspx, https://hofferqst.com/, https://www.iolformula.com/, and https://iolsolver.com/main, respectively). Another investigator (CHY) evaluated the results to determine the plausibility.

### Postoperative refractive outcome analysis

All patients underwent routine postoperative examinations. We assessed the manifest refraction 1 month postoperatively. We adhered to the previously proposed protocols for the postoperative refractive outcome analysis [15, 16], and defined PE as the difference between the spherical equivalent of the postoperative manifest refraction and formula PE using the IOL power implanted. To eliminate systematic error, we zeroed out the mean PE by adjusting the PE for each eye up or down by an amount equal to the mean PE. Negative and positive PEs indicate myopic and hyperopic outcomes, respectively [16]. We defined the absolute prediction error (APE) as the absolute value of PE and calculated the mean value of PE (ME), median of value APE (MedAE), mean value of APE (MAE), and percentages of eyes within ± 0.25 diopter (D), ± 0.50 D, ± 0.75 D, and ± 1.00 D from the target refraction.

### Development of machine learning models to estimate PEs after cataract surgery

We used LightGBM, which was developed by Microsoft and Peking University, to evaluate the relative influence of ocular biometric parameters on the PEs of the nine IOL formulas [17]. LightGBM is one of the most popular machine learning models owing to its superior accuracy, computational speed, and memory consumption compared with those of other machine-learning models [17]. LightGBM was trained using the *lightgbm* library version 3.3.2, with the following hyperparameters: n_estimators = 10,000, learning_rate = 0.01, max_depth = 8, and otherwise, with the default value. We subsequently divided the dataset into development and test sets at an 8:2 ratio and used five-fold cross-validation in the development process. We divided the development set into training and validation sets of 80% and 20%, respectively, for each fold. As a result, the training, validation, and test sets were exclusively constructed at the patient level. We fitted and validated the LightGBM model using the training and validation sets and estimated the PE using the following variables: age, gender, and ocular biometric measurements (including AL, ACD, mean keratometry [K], LT, central corneal thickness [CCT], and horizontal corneal diameter [CD]). We developed five models for the test set to estimate PE for each IOL formula. The predicted values of the five models were averaged for each test sample to determine the final PE using the formula.

### Model performance evaluation and SHAP

We calculated the R squared value (R^2^, coefficient of determination), defined as the proportion of the variation in the dependent variable that can be predicted from the independent variable 1 - RSS/TSS (where RSS is the sum of squares of residuals, and TSS is the total sum of squares), to measure the predictive performance of the models. This value is a measure of how well observed outcomes are replicated by the model based on the proportion of the total variation of outcomes explained by the model. A value of 1 indicates that the model predicts 100% of the relationship, whereas a value of 0.5 indicates that the model predicts 50% of the relationship [18]. We used the bootstrap method to calculate the 95% confidence intervals (CIs) of R^2^. From the test set, the same amount of data as the test set was resampled, with allowance for repetitive samples for the MAE and R^2^ evaluation. This process was repeated 10,000 times to calculate CIs.

We used the SHAP method, a game-theoretic technique used to explain the output of machine learning models, to interpret the model [19]. SHAP values yield quantified contributions, thereby intuitively demonstrating the effect of each feature in terms of the shift of the model output from the base value. SHAP values quantify the effect of individual parameters on the model output and estimated PE. Further details on the SHAP method have been described in the article by Lundberg and Lee [19]. We calculated the SHAP value by determining the average change relative to the presence or absence of individual features after constructing a model with several features. The SHAP value of each feature is an indicator of its strength in terms of positive or negative prediction of the model. A larger absolute SHAP value indicates a greater effect of the feature on the prediction of the model. We calculated the SHAP values to determine the contribution of each variable and their correlation with the PEs of the formulas. Features with positive signs indicate a positive effect on PEs, whereas those with negative signs indicate a negative effect on PEs. The partial dependence plot (PDP) presents the marginal effect of features on the predicted outcome of a machine learning model.

### Development process and analysis

We used Python ver. 3.7.11 (https://www.python.org), *scikit-learn* library ver. 1.0.2, and *shap* library ver. 0.41.0 to develop and analyze the performance of the model. An investigator (RO) performed all developments and inferences on a private server equipped with a central processing unit (CPU) with 32 GB of RAM and an NVIDIA GeForce GTX 3090 with a 24 GB graphics processing unit (GPU; Nvidia). We used *scipy* library ver. 1.7.3 and *scikit_posthocs* library ver. 0.7.0 for statistical analysis. In addition, we used Student’s t-test, Friedman test, post hoc pairwise Wilcoxon test with Holm’s adjustment, Cochrane Q test, and post hoc pairwise Dunn’s test with Holm’s adjustment for comparisons.

## Results

Among the 3,188 eyes of 2,269 patients with cataracts, 1,430 eyes of 1,430 patients (mean age: 69.71 ± 9.14 years, 898 (62.8%) females) were included in this study. Table 1 summarizes the demographic and clinical characteristics of the patients. Table 2 summarizes the predictive performance of the IOL power formulas for postoperative refractive outcomes after zeroing adjustment. The difference in the APE values among the formulas was statistically significant (*P* < 0.05, Friedman test). Supplementary Table 1 presents the results of the pairwise comparison. The Cooke K6 formula exhibited the lowest MAE (0.325) and BUII formula exhibited the lowest MedAE (0.254) values, whereas the SRK/T and Holladay 1 formulas exhibited the highest MAE (0.378) and MedAE (0.307) values, respectively. However, no statistical differences were observed between the Kane, BUII, and Cooke K6 formulas in terms of the MAE value. The Cochrane Q test revealed significant differences between the formulas in terms of the percentages of eyes within the given error range (*P* < 0.001 for all ranges). The Kane formula exhibited the highest percentage of eyes in the given error ranges. Supplementary Table 2 presents the statistical results for the percentage of eyes within the given error range using post hoc Dunn’s test with Holm’s adjustment.

**Table 1:** Demographic and clinical characteristics of subjects

Variables	Value
Number of subjects, Eyes/Patients	1430 / 1430
Age, years	69.71 ± 9.14
Gender, Female	898 (62.8%)
Laterality, Right	782 (54.7%)
AL, mm	23.84 ± 1.25
ACD, mm	3.10 ± 0.43
Flat K, D	43.83 ± 1.47
Steep K, D	44.66 ± 1.52
Corneal Astigmatism, D	0.83 ± 0.59
LT, mm	4.49 ± 0.46
CCT, µm	540.46 ± 32.95
CD, mm	11.72 ± 0.42
IOL Power, D	20.51 ± 2.73
Postoperative Refraction (S.E.), D	-0.47 ± 1.07

ACD, anterior chamber depth measured from corneal epithelium to anterior lens surface; AL, axial length; CCT, central corneal thickness; CD, horizontal corneal diameter; D, diopter; IOL, intraocular lens; K, Keratometry; LT, lens thickness; S.E, spherical equivalent

**Table 2:** Prediction errors for each formula

	ME	SD	MAE	MedAE	± 0.25 D	± 0.50 D	± 0.75 D	± 1.00 D
**BUII**	0.000	0.432	0.328	0.254	49.23%	78.04%	92.73%	97.27%
**Cooke K6**	0.000	0.430	0.325	0.259	48.60%	80.00%	92.45%	97.27%
**EVO V2.0**	0.000	0.438	0.333	0.268	46.78%	78.53%	91.96%	97.13%
**Haigis**	0.000	0.465	0.357	0.289	45.24%	75.45%	89.93%	96.64%
**Hoffer QST**	0.000	0.456	0.350	0.286	44.97%	76.29%	91.19%	96.71%
**Holladay 1**	0.000	0.479	0.369	0.307	42.38%	73.22%	90.00%	96.50%
**Kane**	0.000	0.431	0.326	0.255	49.44%	79.02%	92.73%	97.62%
**PEARL-DGS**	0.000	0.434	0.331	0.264	47.41%	78.32%	92.10%	97.27%
**SRK/T**	0.000	0.494	0.378	0.305	41.61%	72.59%	88.95%	95.45%

D, diopter; ME, mean error; MAE, mean absolute error, MedAE, median absolute error; SD, standard deviation

LightGBM models were constructed to estimate the PEs for each IOL formula. Table 3 summarizes the R^2^ values of the ensemble models for the estimation of PEs using the IOL formulas in the test set. The R^2^ values ranged from 0.021 to 0.231. Notably, the SRK/T and Kane formulas exhibited the highest and lowest R^2^ values in the test set, respectively (R^2^ value: 0.231 and 0.021, respectively). For BUII, Cooke K6, EVO V2.0, and Kane formula, the R^2^ values for the models were not significantly different from zero.

**Table 3:** R^2^ values and their confidence intervals of the models estimating prediction errors in the test set for each formula

	*R* ^2^	95% CI of *R*^2^
**BUII**	0.046	[-0.019, 0.095]
**Cooke K6**	0.025	[-0.009, 0.055]
**EVO V2.0**	0.037	[-0.006, 0.084]
**Haigis**	0.194	[0.113, 0.249]
**Hoffer QST**	0.106	[0.042, 0.176]
**Holladay 1**	0.191	[0.109, 0.266]
**Kane**	0.021	[-0.013, 0.051]
**PEARL-DGS**	0.058	[0.015, 0.093]
**SRK/T**	0.231	[0.132, 0.297]

CI, confidence interval

**Fig. 1:**
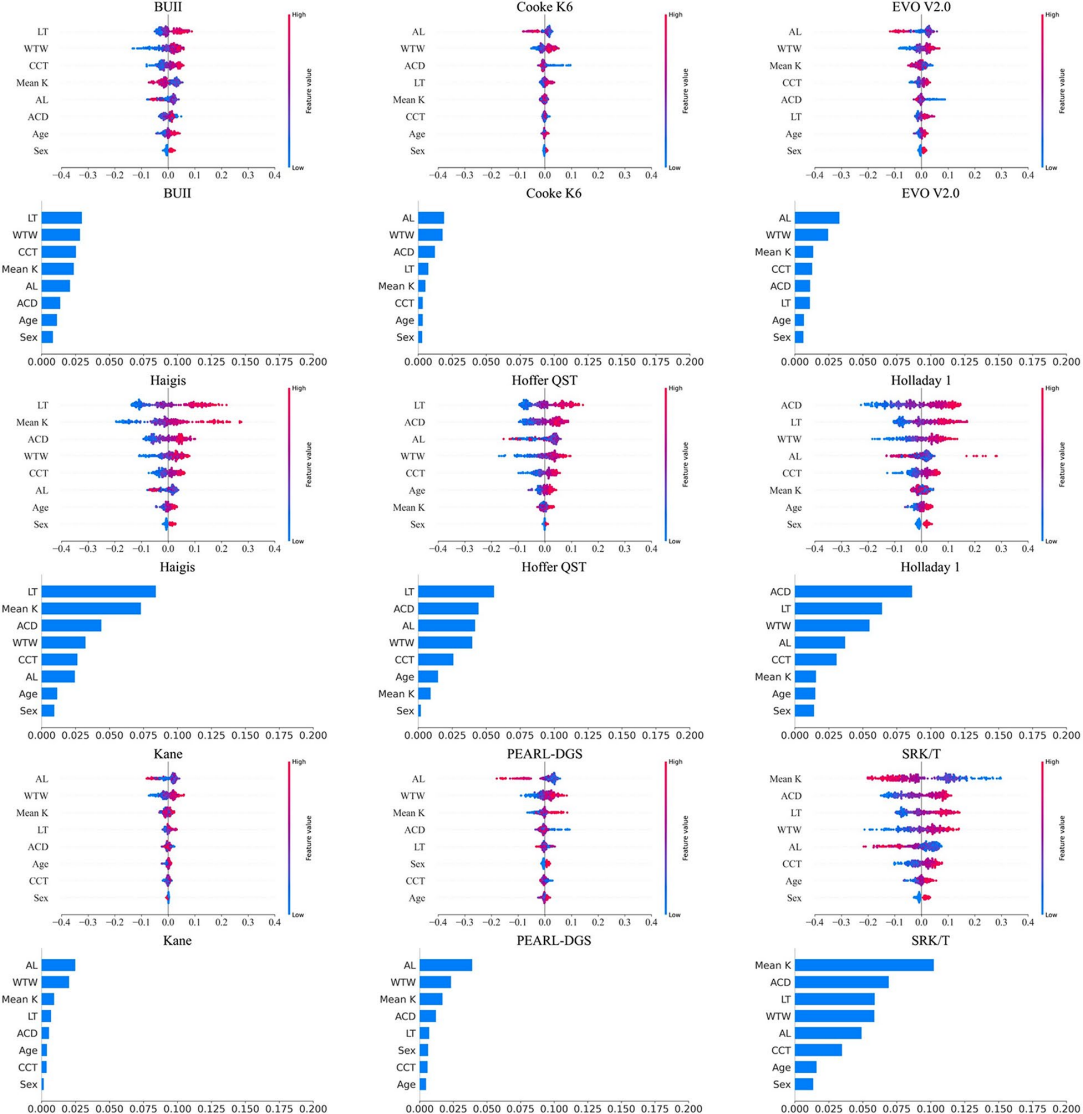
Shapley Additive Explanations (SHAP) values and mean absolute SHAP values for the IOL formulas. ACD, anterior chamber depth measured from corneal epithelium to lens; AL, axial length; BUII, Barrett Universal II; CCT, central corneal thickness; CD, horizontal corneal diameter; IOL, intraocular lens; K, Keratometry; LT, lens thickness. The Shapley additive explanation (SHAP) values and mean absolute SHAP values for each intraocular lens (IOL) formula. The SHAP value for each participant is visually presented as a colored dot. The x-axis represents the SHAP values, wherein negative values correspond to the eyes contributing to a negative prediction error (PE) and positive values correspond to a positive PE. Colors ranging from blue to red denote the value of the ocular biometric variables, with red dots representing eyes with greater values and blue dots representing eyes with smaller values. The average absolute SHAP values for each variable indicate the contribution of the variable variation within the formula. The height of the horizontal bar indicates the contribution of the variable, with larger bars signifying higher contributions and smaller bars indicating lower contributions

The SHAP values are described in Fig. 1. The colored dots represent the SHAP value of each eye. The x-axis represents the SHAP values, wherein the negative values correspond to the eyes contributing to a negative PE and positive values correspond to the eyes contributing to a positive PE. SHAP values of < 0.0 and > 0.0 indicate myopic and hyperopic shifts, respectively. The colors ranging from blue to red represent the values of ocular biometric variables. The red dots represent eyes with greater values, whereas the blue dots represent the eyes with smaller values. The average absolute SHAP values for each variable indicate the contribution of the variation in the variable within the formula. The variable at the top represents the most important variable in the estimation of the PE for each formula. The importance of the variables decreases in descending order.

**Fig. 2:**
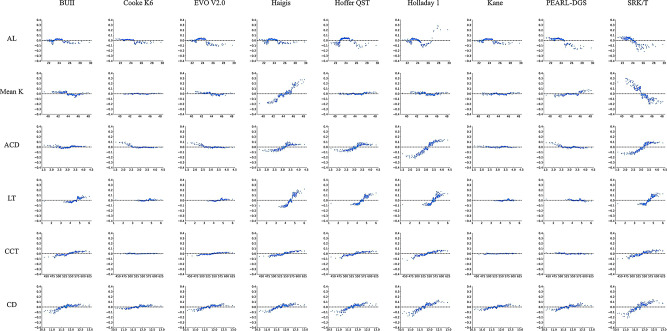
Partial dependence plots (PDPs) between the Shapley Additive Explanations (SHAP) values and ocular biometric variables. ACD, anterior chamber depth measured from corneal epithelium to lens; AL, axial length; BUII, Barrett Universal II; CCT, central corneal thickness; CD, horizontal corneal diameter; IOL, intraocular lens; K, Keratometry; LT, lens thickness. The partial dependence plots (PDPs) between SHAP values and ocular biometric variables present the marginal impact of each variable on each intraocular lens (IOL) formula. The x- and y-axes represent the value of the variable and the SHAP value, respectively. The blue dots represent the eyes. SHAP values of < 0.0 and > 0.0 result in myopic and hyperopic shifts, respectively

A lower R^2^ value of the IOL formula indicated a smaller absolute SHAP value. Figure 2 presents the PDPs between the SHAP values and ocular biometric variables. The x- and y-axes represent the value of the variable and the SHAP value, respectively. The SHAP values in the figures represent the marginal impact of the variables on the PEs based on the presence or absence of other variables. A longer AL was independently associated with more myopic PE in the SRK/T formula. The remaining formulas showed irregular trends. A steeper K results in myopic PE in the SRK/T formula, whereas it leads to hyperopic PEs in the Haigis formula. All variables exhibited a nearly flat curve in the PDP plots for the Kane formula, indicating that the performance efficiency of the formula was less affected by these variables.

## Discussion

We developed machine learning models to predict PEs in cataract surgery in this study. The R^2^ values were generally low, ranging from 0.021 to 0.232. The SRK/T formula exhibited the highest R^2^ value for estimating PE, whereas the BUII, Cooke K6, EVO V2.0, and Kane formula exhibited the lowest R^2^ value, which did not statistically differ from zero. These findings indicate that ocular biometric parameters, including AL, ACD, K, LT, CCT, and CD, have significant effects on the PE of the SRK/T formula. However, they had no significant effect on the PE of the BUII, Cooke K6, EVO V2.0, and Kane formula. To the best of our knowledge, this is the first study to use ocular biometric variables to estimate PEs using machine learning.

Previous studies have evaluated the accuracy of IOL power formulas for specific subgroups of patients according to the ocular biometric variables [6, 7, 20–28] and provided valuable insights into the strengths and weaknesses of various formulas. The Kane formula exhibits a significantly lower MAE value and higher accuracy in eyes with long AL. Moreover, it outperforms other formulas, including the Hill-RBF 2.0 and BUII formulas, in eyes with short AL [12, 29]. Several studies have used other ocular biometric parameters in combination with AL. Kim et al. categorized eyes based on the AL, K, and ACD values and compared the accuracies of the Haigis, Hoffer Q, Holladay 1, SRK/T, and BUII formulas in each subgroup [14]. Hipólito-Fernandes et al. revealed that the Kane, PEARL-DGS, and EVO V.2.0 formulas yielded reliable and stable results in subgroups with extreme combinations of ACD and LT [30]. Although previous studies have provided valuable insights into ocular biometric combinations, an optimal formula for each subgroup of eyes remains to be established.

The ocular biometric parameters AL, K, ACD, LT, WTW, and CCT showed significant associations with each other. Kim et al. conducted a large-scale ocular biometric analysis and revealed that AL was positively correlated with ACD, WTW, and CCT and negatively correlated with K and LT [14]. Another larger study revealed similar tendencies among AL, ACD, K, and LT [31]. Furthermore, these parameters also show associations with age [14, 31–34]. Thus, subgroup analysis using individual variables introduced significant confounding factors, given the inherent correlations among the ocular biometric parameters. The effects of these confounding factors cannot be adequately addressed in studies using such designs. Multivariate analysis must be conducted to predict PEs and identify the best-fitting formula owing to the interdependence of these variables.

We incorporated nine ocular biometric parameters to estimate PEs using machine learning models. Traditional regression models consider all relationships as linear, thereby limiting their ability to evaluate complex interactions between variables. In contrast, machine learning models incorporate nonlinear relationships, thereby enhancing their predictive capabilities. However, the mechanisms underlying machine learning models are challenging owing to their complex structures. The SHAP method addresses this limitation by aiding in the interpretation of the outcomes of machine learning models. Moreover, it enables the exploration of the importance and dependence of variables.

The machine learning models produced R² scores below 30% for all formulas, suggesting that at least 70% of the variability cannot be explained by the ocular biometric variables included in this study. The machine learning model exhibited the lowest R^2^ score of 0.021 for the Kane formula. The machine learning models for BUII, Cooke K6, EVO V2.0, and Kane formula failed to introduce significant performance in estimating the variability in the predicted PE, indicating their stability across ocular biometric parameter ranges. In contrast, the R^2^ scores of the third-generation formulas, SRK/T, Haigis, and Holladay 1, were relatively high, indicating a stronger association between the predicted PEs and ocular biometric variables.

The impact of each variable on the prediction of PE can be discerned (Fig. 1). Notably, the absolute SHAP values of the BUII, Cooke K6, EVO V2.0, and Kane formulas were lower than those of the Haigis, Holladay 1, and SRK/T formulas. The PDP plots illustrate the adjusted influence of each variable while considering the influence of the other variables. Thus, these graphs depict the response of different formulas to changes in ocular biometry. An increase in AL leads to the SRK/T formula predicting a negative PE, whereas smaller K, larger ACD, and LT values lead to the SRK/T formula predicting greater PEs. In contrast, LT and K, rather than AL, exhibit significant effects on the Haigis formula.

The findings of our study highlight different aspects of the objectives and designs of numerous previous studies that attempted to differentiate subgroups based on the range of ocular biometric parameters and compared various formulas within these subgroups to identify the best formula in terms of PE or APE. However, the outcomes varied among research groups. Moreover, the differences often failed to demonstrate statistical differences in subgroup analyses. The findings of our study can be used to understand these divergent findings. New-generation formulas, such as the BUII, Cooke K6, EVO V2.0, and Kane, consistently yielded stable results across a wide range of parameters. In contrast, the SRK/T, Holladay 1, and Haigis formulas exhibited a less consistent performance in the subgroup analysis, which may be attributed to the PEs being explainable using known ocular biometric variables.

Our study demonstrated that the new-generation formulas are generally stable across a wide range of known ocular biometric parameters. We suggest that further research should focus not on the previous known ocular biometric parameters but on the new variables which can have affect on the PEs, such as the lens vault, angle kappa, and accuracy of the ocular biometric measurements [23, 35, 36]. New variables using the mathematical combination of the known variables, such as AL/corneal radius [37], might have a role in the change of the PEs.

This study has some limitations. First, our sample size was relatively small for the machine learning model. However, a sample size exceeding 1000 is sufficient to demonstrate this tendency. Further large-scale studies could reveal a higher proportion of potentially predictable variance. Second, we only included a single type of IOL in this study; thus, the findings of our study must be validated before being applied to other IOLs. Lastly, the machine learning model for each formula could be overfitted to our dataset, which could have resulted in bias. Moreover, comparisons have been made between these overfitted models, but not with an ideal prediction algorithm.

In conclusion, machine learning algorithms can predict a portion of PEs; however, they have limitations in explaining the variability of PEs using ocular biometric parameters. Newer IOL formulas, such as the BUII, Cooke K6, EVO V2.0, and Kane formulas, demonstrated relatively stable outcomes across a wide range of ocular biometric variables, indicating that these formulas are already optimized by ocular biometric variables used in this study. Introducing additional ocular biometric variables beyond those currently used in the IOL calculation formula may improve the accuracy of future surgical results.

## Electronic supplementary material

Below is the link to the electronic supplementary material.

Supplementary Material 1

## Data Availability

The datasets used and analyzed during the current study are available from the corresponding author on reasonable request.

## Declarations

### Meeting presentation

16th Joint Meeting of Korea-China-Japan Ophthalmologists, 2023.

## Abbreviations

ACD: anterior chamber depth measured from corneal epithelium to anterior lens surface
AL: axial length
APE: absolute prediction error
CCT: central corneal thickness
CD: horizontal corneal diameter
D: diopter
DM: diabetes mellitus
HTN: hypertension
IOL: intraocular lens
K: keratometry
LT: lens thickness
ME: mean prediction error
MAE: mean absolute prediction error
MedAE: median absolute prediction error
PE: prediction error
SD: standard deviation
SE: spherical equivalent
