# Supplementary material for: Evaluation of prediction errors in nine intraocular lens calculation formulas using an explainable machine learning model
Source: BMC Ophthalmol. 2024 Dec 19;24:531. doi: 10.1186/s12886-024-03801-2 (PMC11657498; doi:10.1186/s12886-024-03801-2)
Supplement: Supplementary file 1 — Supplementary Material 1 [file 12886_2024_3801_MOESM1_ESM.docx]

Supplementary Table 1. Pairwise comparison of absolute prediction error between six intraocular lens power calculation formulas after zeroing adjustment of prediction error

| **APE** | **BUII** | **Cooke K6** | **EVO V2.0** | **Haigis** | **Hoffer QST** | **Holladay 1** | **Kane** | **PEARL-DGS** |
| --- | --- | --- | --- | --- | --- | --- | --- | --- |
| **Cooke K6** | >0.999 |  |  |  |  |  |  |  |
| **EVO V2.0** | >0.999 | 0.112 |  |  |  |  |  |  |
| **Haigis** | <0.001 | <0.001 | <0.001 |  |  |  |  |  |
| **Hoffer QST** | <0.001 | <0.001 | <0.001 | >0.999 |  |  |  |  |
| **Holladay 1** | <0.001 | <0.001 | <0.001 | >0.999 | 0.006 |  |  |  |
| **Kane** | >0.999 | >0.999 | >0.999 | <0.001 | <0.001 | <0.001 |  |  |
| **PEARL-DGS** | >0.999 | 0.036 | >0.999 | <0.001 | 0.004 | <0.001 | >0.999 |  |
| **SRK/T** | <0.001 | <0.001 | <0.001 | 0.155 | <0.001 | 0.199 | <0.001 | <0.001 |

APE = absolute prediction error

Supplementary Table 2. Statistical results for the percentage of eyes within the given error range after zeroing adjustment of prediction error using post-hoc Dunn’s test with Bonferroni adjustment.

| **APE < 0.25 D** |  |  |  |  |  |  |  |  |
| --- | --- | --- | --- | --- | --- | --- | --- | --- |
|  | **BUII** | **Cooke K6** | **EVO V2.0** | **Haigis** | **Hoffer QST** | **Holladay 1** | **Kane** | **PEARL-DGS** |
| **Cooke K6** | >0.999 |  |  |  |  |  |  |  |
| **EVO V2.0** | >0.999 | >0.999 |  |  |  |  |  |  |
| **Haigis** | 0.554 | >0.999 | >0.999 |  |  |  |  |  |
| **Hoffer QST** | 0.748 | >0.999 | >0.999 | >0.999 |  |  |  |  |
| **Holladay 1** | 0.008 | 0.026 | 0.471 | >0.999 | >0.999 |  |  |  |
| **Kane** | >0.999 | >0.999 | >0.999 | 0.442 | 0.586 | 0.005 |  |  |
| **PEARL-DGS** | >0.999 | >0.999 | >0.999 | >0.999 | >0.999 | 0.194 | >0.999 |  |
| **SRK/T** | 0.002 | 0.006 | 0.160 | >0.999 | >0.999 | >0.999 | 0.001 | 0.056 |
|  |  |  |  |  |  |  |  |  |
| **APE < 0.50 D** |  |  |  |  |  |  |  |  |
|  | **BUII** | **Cooke K6** | **EVO V2.0** | **Haigis** | **Hoffer QST** | **Holladay 1** | **Kane** | **PEARL-DGS** |
| **Cooke K6** | >0.999 |  |  |  |  |  |  |  |
| **EVO V2.0** | >0.999 | >0.999 |  |  |  |  |  |  |
| **Haigis** | >0.999 | 0.471 | >0.999 |  |  |  |  |  |
| **Hoffer QST** | >0.999 | 0.103 | >0.999 | >0.999 |  |  |  |  |
| **Holladay 1** | 0.060 | 0.001 | 0.022 | >0.999 | >0.999 |  |  |  |
| **Kane** | >0.999 | >0.999 | >0.999 | >0.999 | 0.548 | 0.008 |  |  |
| **PEARL-DGS** | >0.999 | >0.999 | >0.999 | >0.999 | >0.999 | 0.034 | >0.999 |  |
| **SRK/T** | 0.016 | <0.001 | 0.005 | 0.471 | >0.999 | >0.999 | 0.002 | 0.009 |
|  |  |  |  |  |  |  |  |  |
| **APE < 0.75 D** |  |  |  |  |  |  |  |  |
|  | **BUII** | **Cooke K6** | **EVO V2.0** | **Haigis** | **Hoffer QST** | **Holladay 1** | **Kane** | **PEARL-DGS** |
| **Cooke K6** | >0.999 |  |  |  |  |  |  |  |
| **EVO V2.0** | >0.999 | >0.999 |  |  |  |  |  |  |
| **Haigis** | >0.999 | >0.999 | >0.999 |  |  |  |  |  |
| **Hoffer QST** | 0.243 | 0.451 | >0.999 | >0.999 |  |  |  |  |
| **Holladay 1** | 0.276 | 0.520 | >0.999 | >0.999 | >0.999 |  |  |  |
| **Kane** | >0.999 | >0.999 | >0.999 | >0.999 | 0.243 | 0.276 |  |  |
| **PEARL-DGS** | >0.999 | >0.999 | >0.999 | >0.999 | 0.944 | >0.999 | >0.999 |  |
| **SRK/T** | 0.012 | 0.030 | 0.136 | 0.835 | >0.999 | >0.999 | 0.012 | 0.092 |
|  |  |  |  |  |  |  |  |  |
| **APE < 1.00 D** |  |  |  |  |  |  |  |  |
|  | **BUII** | **Cooke K6** | **EVO V2.0** | **Haigis** | **Hoffer QST** | **Holladay 1** | **Kane** | **PEARL-DGS** |
| **Cooke K6** | >0.999 |  |  |  |  |  |  |  |
| **EVO V2.0** | >0.999 | >0.999 |  |  |  |  |  |  |
| **Haigis** | >0.999 | >0.999 | >0.999 |  |  |  |  |  |
| **Hoffer QST** | >0.999 | >0.999 | >0.999 | >0.999 |  |  |  |  |
| **Holladay 1** | >0.999 | >0.999 | >0.999 | >0.999 | >0.999 |  |  |  |
| **Kane** | >0.999 | >0.999 | >0.999 | >0.999 | >0.999 | >0.999 |  |  |
| **PEARL-DGS** | >0.999 | >0.999 | >0.999 | >0.999 | >0.999 | >0.999 | >0.999 |  |
| **SRK/T** | 0.182 | 0.182 | 0.316 | >0.999 | >0.999 | >0.999 | 0.031 | 0.182 |

APE = absolute prediction error
